# Changes in prevalence and risk factors of hypertension among adults in Bangladesh: An analysis of two waves of nationally representative surveys

**DOI:** 10.1371/journal.pone.0259507

**Published:** 2021-12-02

**Authors:** Muhammad Abdul Baker Chowdhury, Mirajul Islam, Jakia Rahman, Mohammed Taj Uddin, Md Rabiul Haque, Md Jamal Uddin

**Affiliations:** 1 Department of Neurosurgery, College of Medicine, University of Florida, Gainesville, Florida, United States of America; 2 Department of Statistics, University of Florida, Gainesville, Florida, United States of America; 3 Department of Statistics, University of Dhaka, Dhaka, Bangladesh; 4 Department of Mathematics and Natural Sciences, BRAC University, Dhaka, Bangladesh; 5 Department of Statistics, Shahjalal University of Science & Technology, Sylhet, Bangladesh; 6 Department of Population Sciences, University of Dhaka, Dhaka, Bangladesh; University of Southern Queensland, AUSTRALIA

## Abstract

**Introduction:**

Bangladesh is one of the countries where the prevalence of non-communicable diseases (NCDs) such as hypertension is rising due to rising living standards, sedentary lifestyles, and epidemiological transition. Among the NCDs, hypertension is a major risk factor for CVD, accounting for half of all coronary heart disease worldwide. However, detailed research in this area has been limited in Bangladesh. The objective of the study was to estimate changes in the prevalence and risk factors of hypertension among Bangladeshi adult population. The study also sought to identify socioeconomic status-related inequality of hypertension prevalence in Bangladesh.

**Methods:**

Cross-sectional analysis was conducted using nationally representative two waves of the Bangladesh Demographic and Health Survey (BDHS) in 2011 and 2017–18. Survey participants were adults 18 years or older- which included detailed biomarker and anthropometric measurements of 23539 participants. The change in prevalence of hypertension was estimated, and adjusted odds ratios were obtained using multivariable survey logistic regression models. Further, Wagstaff decomposition method was also used to analyze the relative contributions of factors to hypertension.

**Results:**

From 2011 to 2018, the hypertension prevalence among adults aged ≥35 years increased from 25.84% to 39.40% (p<0.001), with the largest relative increase (97%) among obese individuals. The prevalence among women remained higher than men whereas the relative increase among men and women were 75% and 39%, respectively. Regression analysis identified age and BMI as the independent risk factors of hypertension. Other risk factors of hypertension were sex, marital status, education, geographic region, wealth index, and diabetes status in both survey years. Female adults had significantly higher hypertension risk in both survey years in the overall analysis in, however, in the subgroup analysis, the gender difference in hypertension risk was not significant in rural 2011 and urban 2018 samples. Decomposition analysis revealed that the contributions of socio-economic status related inequality of hypertension in 2011 were46.58% and 20.85% for wealth index and BMI, respectively. However, the contributions of wealth index and BMI have shifted to 12.60% and 55.29%, respectively in 2018.

**Conclusion:**

The prevalence of hypertension among Bangladeshi adults has increased significantly, and there is no subgroup where it is decreasing. Population-level approaches directed at high-risk groups (overweight, obese) should be implemented thoroughly. We underscore prevention strategies by following strong collaboration with stakeholders in the health system of the country to adopt healthy lifestyle choices.

## Introduction

Hypertension is the most significant risk factor for cardiovascular disease (CVD), leading to fifty percent of the coronary heart disease and nearly two-thirds of the burden of cerebrovascular disease worldwide [1, 2]. It is also associated with other diseases, such as stroke, kidney failure, disability, and premature death [3, 4]. An estimation of the World Health Organization (WHO), suggests that 1.13 billion people worldwide had hypertension in 2019, and two-thirds of them were living in low- and middle-income countries (LMICs) [5]. A study reported that the relationship between hypertension and CVD is stronger in low-income Asian countries than in developed countries [6]. Globally, every year approximately one third of the total deaths are caused by CVD [7]. With the rapid increase in population aging, urbanization, and overweight/obesity, the prevalence of hypertension in Asian countries continues to rise [6, 8, 9]. The increasing burden of hypertension in Asian and other LMICs has been attributed to the epidemiological transition, which is characterized by escalating non-communicable diseases (NCDs) and decreasing infectious diseases [10–12].

Bangladesh a low- and middle-income Asian country is experiencing an epidemiological transition from communicable to non-communicable diseases [13]. The country is also experiencing a nutritional transition, from a traditional dietary practice to a fast-food diet and sedentary lifestyle due to an improved socio-economic status and unplanned rapid urban development where people are living in a congested area with less physical activity [13]. Furthermore, an increase in life expectancy due to better healthcare services is also contributing to the higher prevalence of NCDs, such as hypertension as older individuals are more prone to have a higher prevalence of NCDs [14–16].

The prevalence of hypertension among Bangladeshi adults ages 35 years and above was 26.4% in 2011, which is around 25 times higher than that was in 1976 [17, 18]. Previous studies inevitably confirm that hypertension prevalence in Bangladesh varied widely depending on the study characteristics, study settings/design, or target population; however, all findings confirm that hypertension prevalence among adults is on the rise [19–21]. The available studies on hypertension in Bangladesh predominantly focused on a specific group of population such as urban or rural individuals, or slum residents, or hospital-based studies [17, 18, 22, 23]. While an upward trend in the prevalence of hypertension is evident, a very few population-based studies also reported the prevalence of hypertension, which are outdated [20, 24, 25]. Despite the growing literature of hypertension in Bangladesh, there is no study reporting the changes in prevalence of hypertension and its associated factors or studied comparison of its risk factors. Moreover, to combat the rising prevalence of NCDs, the health system of Bangladesh is facing challenges in absence a population-based surveillance system to track the burden of these diseases including hypertension [15, 26]. Therefore, examining the changes in hypertension prevalence and its associated factors in Bangladesh is paramount. Indeed, understanding the changes in hypertension prevalence risk factors for different subgroups will contribute to the targeted efforts to improve hypertension disease management in the long term.

In this serial cross-sectional study, our purposes are to 1) estimate the changes in prevalence of hypertension among adults in Bangladesh using most recent national survey data from 2011 and 2018, 2) explore the factors that potentially contribute to the risk of hypertension among the studied population and make a comparison among them, 3) we also examined the inequality in hypertension among adults and decomposed the factors behind this.

## Methods

### Study population and data source

We used the Bangladesh Demographic and Health Survey (BDHS), de-identified and publicly available datasets [27, 28]. We included data from two waves of the BDHS, i.e., 2011 and 2017–18 (written as 2018 onward), which are the latest available data [27, 28]. The BDHS surveys are conducted in three years cycles since 1993, however, biomarker information was collected only in these two years. The study population for both surveys was recruited from the entire country using multistage cluster sampling. The details of the sampling methods and survey protocols for both surveys have been reported elsewhere [27, 28]. In brief, the 2018 BDHS, biomarkers and relevant information were collected from all women and men age 18 and older; one in four of the households selected for the survey. Total eligible adult participants were 14704 (8013 women and 6691 men). Similarly, in the 2011 BDHS, biomarkers and relevant information were collected from all women and men age 35 and older; in one three of the households selected for the survey and the total eligible participants were 8835 (4312 women and 4523 men). We followed the Strengthening the Reporting of Observational Studies in Epidemiology (STROBE) reporting guideline.

### Measurement of blood pressure

Blood pressure (BP) was measured using the WHO standard protocol. Three measurements of systolic and diastolic BP were taken during the interview using a digital oscillometer device with automatic upper-arm inflation and an automatic pressure release. There were at least 5 minutes interval between each measurement. The average of second and third measurement was used to report. The optimal BP was defined as SBP <120 and DBP <80 mm Hg without antihypertensive medication use. Normal BP was defined by SBP of 120 to 129 mm Hg with DBP 80–84 mm Hg without antihypertensive medication use. High normal BP was defined as SBP of 130–139 mm Hg and DBP of 85–89 mm Hg without antihypertensive medication use. The Grade 1 hypertension or mildly elevated hypertension was defined as SBP 140 to 159 mm Hg with DBP 90–99 mm Hg, Grade 2 hypertension or moderately elevated hypertension was defined as SBP 160–179 or DBP 100–109 mm Hg, and Grade 3 hypertension or severely elevated hypertension was defined as SBP 180+ or DBP 110+ mm Hg. For our analysis we created a dichotomies hypertension variable using SBP ≥140 or DBP ≥90 mm Hg or antihypertensive medication use as 1 or 0 otherwise.

### Covariates

Demographic variables included in this analysis were participant’s age (grouped into 18–24, 25–29, 30–34, 35–44, 45–54, 55–64, 65–74, and 75+ years of age), sex, marital status (currently married, not currently married), place of residence (urban, rural), geographic region (division of residence), educational level (no education, primary, secondary, higher), wealth index based on principal component analysis (poorest, poorer, middle, richer, richest), working status (yes, no), diabetes status (yes, no), and body mass index (BMI). The BMI was calculated as weight in kilograms divided by height in meters squared. We used BMI classifications for Asian population: normal weight (18.5 to 23.0), moderate risk/ overweight (23.0 to <27.5), high risk/obese (≥27.5) [29]. A person was classified as having diabetes if his/her fasting plasma glucose value was of 7.0 mmol/L or above, or taking anti-diabetic medication [30].

### Statistical analysis

Descriptive analysis of demographic and socioeconomic characteristics was performed using full sample of each survey. We compared the distribution of these characteristics among participants by hypertension status and survey periods using proportion test. We also compared the proportion of hypertension by survey years and calculated the relative increase between the survey periods. To predict the risk of having hypertension we used multiple logistic regression models after controlling for potential covariates. Since all the covariates were found to have significant associations with hypertension status in bivariate analysis, they were all included in the multivariable model. We estimated Variance Inflation Factor (VIF) to check for multicollinearity with a cut off value of 10.

To make an appropriate comparison between the two study periods, firstly we compared regression estimates of 2011 and 2018 BDHS data of the study participants aged 35 years and older. Then we separately analyzed 18–34 years study participants in the 2018 BDHS survey to estimate the factors that predict the risk of hypertension among younger adults. For all analyses, the statistical significance was set at 2-sided (p-value < 0.05). We performed all data management and statistical analyses using Stata 15 (StataCorp, College Station, TX, USA). To account for complex survey design, we considered the sample weights, primary sampling units, and Strata using “SVY” command of Stata. The “svysubpop” command was used for subgroup analyses.

### Concentration index

The concentration index represents the magnitude of inequality by estimating the area between the concentration curve and line of equality. It is calculated as two times the weighted covariance between the outcome and fractional rank in the wealth distribution divided by the mean of the variable. The concentration index can be written as follows:

$$C=\frac{2}{\mu }cov(y_{i},R_{i})$$

where C, *R*_*i*_ and *y*_*i*_ denote the concentration index, the fractional rank of i^th^ individual in the distribution of socioeconomic position and the outcome variable index, respectively; and μ is the mean of the outcome variable of the sample [31].

### Decomposition of concentration index

We further decomposed the concentration index to determine the relative impact of various socio-economic determinants of hypertension among adults. To do this, we used regression-based decomposition technique proposed by Wagstaff et al.[32]. The Wagstaff’s method demonstrates that the concentration index can be decomposed into the contributions of each factor to the income-related inequalities [32]. Each contribution is determined by the sensitivity of outcome to that socioeconomic component as well as the degree of income-related inequality in that factor.

### Ethics

The BDHS surveys including anthropometric (height, and weight) and biomarkers were approved by the institutional review boards of the ICF International and Bangladesh Medical Research Council. Written informed consent was taken from the all study participants.

## Results

### Changes in hypertension prevalence

Table 1 shows the distribution of study participants aged ≥ 35 years by socio-demographic characteristics and hypertension prevalence in 2011 and 2018. From 2011 to 2018, the hypertension prevalence among men and women 35 years or older increased from 25.84% to 39.40% (p<0.001), a 52% relative increase. During this period, the prevalence of hypertension increased from 19.53% to 34.03% in men and from 31.97% to 44.5% in female, with relative increase of 74% and 39%, among men and women, respectively. Across all population groups, increase in the prevalence of hypertension are highly statistically significant. Age-specific prevalence of hypertension increased significantly in all age groups with 63% highest relative increase among 35–44-year-old adults. The highest prevalence of hypertension among ≥75 years has increased from 41.66% to 59.85% during this period. Similarly, the prevalence of hypertension has increased in both males and females. There was much larger relative increase (60%) in prevalence of hypertension among married adults. Interestingly, prevalence of hypertension among adults with no education increased from 26.57% to 39.19% over time. The highest relative prevalence of hypertension was 67% and 64% among adults with primary and secondary education, respectively.

**Table 1:** Sociodemographic characteristics of adults age 35 years and older and hypertension rate in Bangladesh, 2011–2018.

Variables	Distribution 2011–2018, %	Hypertension 2011 BDHS, % (SE)	Hypertension 2018 BDHS, % (SE)	p-value difference	Ratio
All adults age 35 years and older	100%	25.84 (0.0067)	39.40 (0.0071)	<0.001	1.52
Age group					
35–44	36.01	17.46 (0.0087)	28.45 (0.0095)	<0.001	1.63
45–54	27.44	24.90 (0.0114)	38.48 (0.0148)	<0.001	1.55
55–64	18.66	30.05 (0.0152)	46.75 (0.0140)	<0.001	1.56
65–74	11.26	39.30 (0.0181)	51.46 (0.019)	<0.001	1.31
75+	6.63	41.66 (0.0236)	59.85 (0.0259)	<0.001	1.44
Sex					
Male	49.01	19.53 (0.008)	34.03 (0.0099)	<0.001	1.74
Female	50.99	31.97 (0.0093)	44.5 (0.0092)	<0.001	1.39
Marital status					
Not married	15.85	41.08 (0.0155)	53.7 (0.0155)	<0.001	1.31
Married	84.15	22.99 (0.0068)	36.69 (0.0076)	<0.001	1.6
Educational level					
No education	44.72	26.57 (0.0092)	39.19 (0.0108)	<0.001	1.47
Primary	29.13	22.28 (0.0106)	37.26 (0.0113)	<0.001	1.67
Secondary	18.07	25.87 (0.0138)	42.47 (0.0153)	<0.001	1.64
Higher	8.08	33.59 (0.0204)	41.95 (0.0212)	0.0004	1.25
Place of residence					
Urban	24.31	32.67 (0.0127)	42.93 (0.0134)	<0.001	1.31
Rural	75.69	23.76 (0.0076)	38.21 (0.0083)	<0.001	1.61
Geographic region					
Barisal	5.80	24.58 (0.0158)	46.0 (0.0194)	<0.001	1.87
Chittagong	16.54	22.16 (0.0147)	42.46 (0.0204)	<0.001	1.92
Dhaka	31.72	27.19 (0.0141)	35.65 (0.014)	<0.001	1.31
Khulna	13.37	29.69 (0.0181)	40.64 (0.0178)	<0.001	1.37
Rajshahi	14.3	24.22 (0.015)	38.55 (0.0181)	<0.001	1.59
Rangpur	12.34	28.18 (0.017)	41.23 (0.0161)	<0.001	1.46
Sylhet	5.92	20.35 (0.019)	39.08 (0.0211)	<0.001	1.92
Wealth index					
Poorest	19.77	18.92 (0.0124)	34.48 (0.0141)	<0.001	1.82
Poorer	19.72	21.79 (0.0125)	34.23 (0.0151)	<0.001	1.57
Middle	20.18	22.62 (0.013)	38.98 (0.015)	<0.001	1.72
Richer	19.61	27.8 (0.0126)	40.76 (0.017)	<0.001	1.47
Richest	20.73	37.09 (0.0136)	48.55 (0.0141)	<0.001	1.31
Body Mass Index					
Underweight	19.70	18.4 (0.0113)	25.9 (0.0134)	<0.001	1.41
Normal weight	36.27	22.94 (0.0097)	33.92 (0.0098)	<0.001	1.48
Overweight	20.4	41.09 (0.017)	47.98 (0.0122)	0.0001	1.17
Obese	23.63	27.96 (0.0099)	54.99 (0.0177)	<0.001	1.97
Currently working					
No	43.53	33.23 (0.0094)	49.49 (-0.0109)	<0.001	1.49
Yes	56.47	17.76 (0.0077)	34.11 (-0.0085)	<0.001	1.92
Diabetes					
No	84.99	24.26 (0.3866)	37.56 (-0.0077)	<0.001	1.55
Yes	15.01	38.66 (0.0202)	47.11 (-0.016)	<0.001	1.22

**Fig 1:**
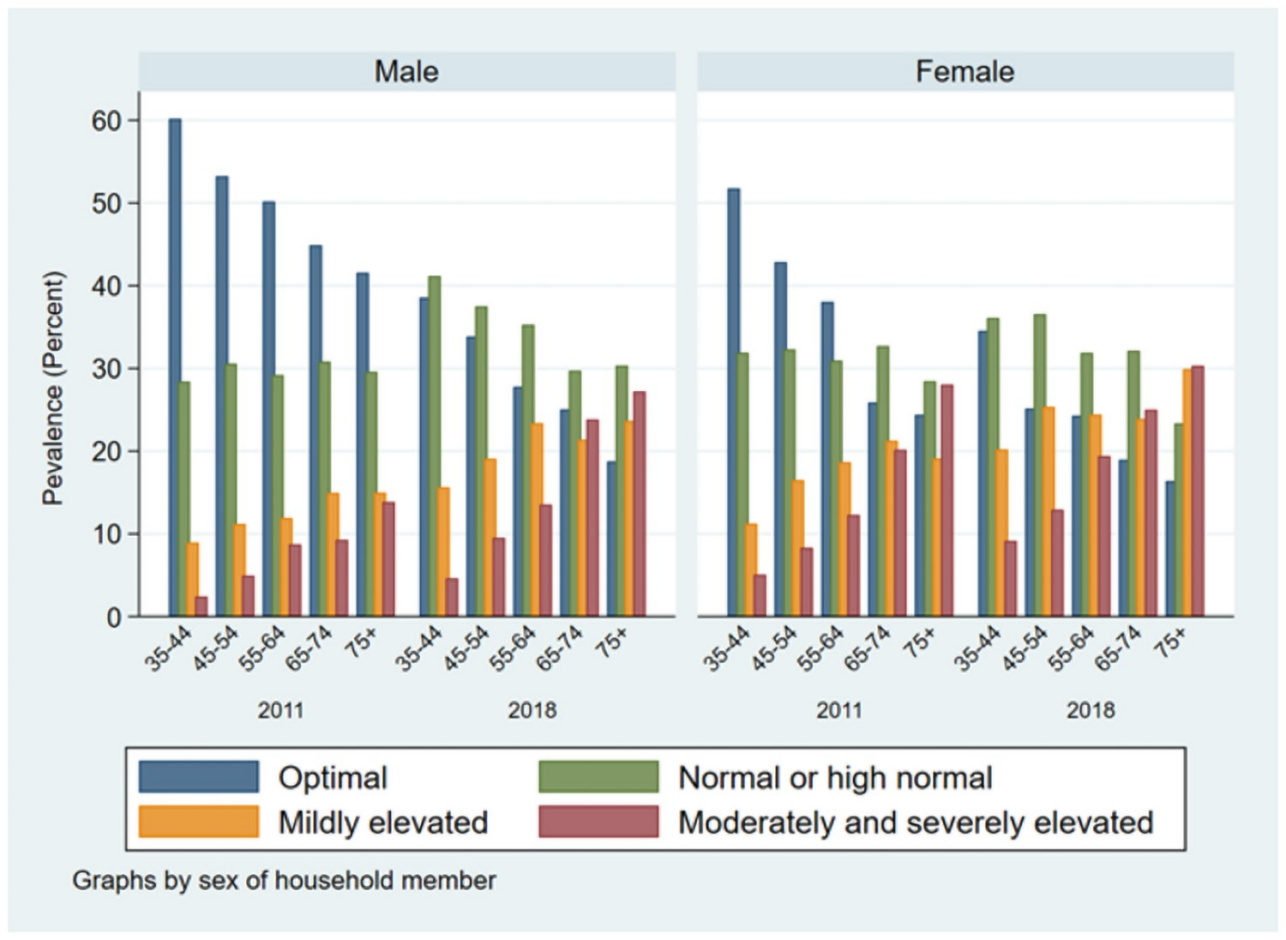
Blood pressure categories by age, sex, and survey year.

**Fig 2:**
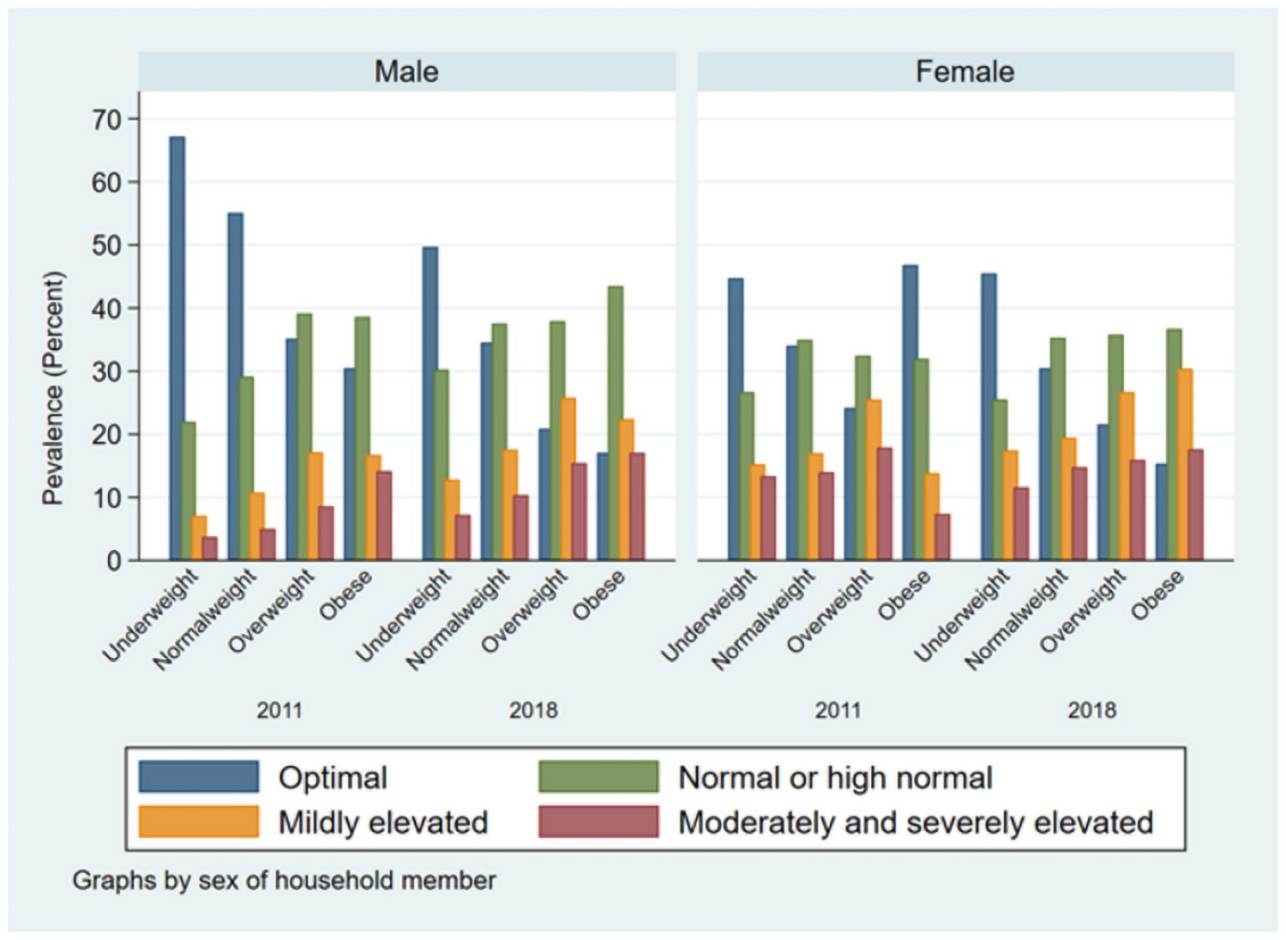
Blood pressure categories by BMI, sex, and survey year.

Overtime the prevalence of hypertension increased significantly both in urban and rural areas, with a relative change of 61% in rural areas. There was proportionate significant increase in hypertension prevalence in all geographic locations (division) of Bangladesh, especially the prevalence doubled among study participants from Sylhet division (20.35% to 39.08%, p<0.001) from 2011 to 2018. The increase in prevalence of hypertension was 82% among the lowest socio-economic individuals, followed by 72% in middle, and 57% second-lowest socio-wealth groups during this period. The prevalence of hypertension increased two-folds among obese adults; remarkably the prevalence also significantly increased among underweight adults from 18% in 2011 to 26% in 2018. Hypertension prevalence also increased significantly by 55% and 22% among diabetic and non-diabetic adults, respectively. Figs 1 and 2 show the blood pressure categories by survey year, gender, and BMI categories. It appears that moderate to severely elevated blood pressure is highly correlated with higher BMI and increasing age in both survey years.

### Risk factors of hypertension (≥35 years adults)

The results of the multiple logistic regression suggest that age, sex, marital status, education, geographic region, wealth index, BMI, and diabetes had significant effects on hypertension in both 2011 and 2018 (Table 2). Working status was a significant predictor of hypertension in 2011 but in 2018 it had no significant effect (AOR: 0.90, 95% CI: 0.78, 1.05). The highest risk of having hypertension was found in age group ≥75 in both surveys than the younger age groups (35–44 years).

**Table 2:** Adjusted odds ratios of potential factors associated with hypertension among adults age 35 and older in Bangladesh, BDHS 2011–2018.

	2011 BDHS		2017–18 BDHS	
Variables	OR (95% CI)	p-value	OR (95% CI)	p-value
Age group				
35–44	Ref		Ref	
45–54	1.85 (1.56, 2.19)	<0.001	1.75 (1.49, 2.05)	<0.001
55–64	2.48 (2.01, 3.05)	<0.001	2.78 (2.38, 3.23)	<0.001
65–74	4.04 (3.24, 5.05)	<0.001	3.63 (2.96, 4.46)	<0.001
75+	4.27 (3.24, 5.63)	<0.001	4.99 (3.77, 6.60)	<0.001
Sex				
Male	Ref		Ref	
Female	1.42 (1.1, 1.82)	0.006	1.46 (1.26, 1.69)	<0.001
Marital status				
Not married	Ref		Ref	
Married	0.72 (0.61, 0.85)	<0.001	0.81 (0.68, 0.96)	0.014
Educational level				
No education	Ref		Ref	
Primary	0.88 (0.75, 1.03)	0.111	1.12 (0.97, 1.28)	0.117
Secondary	1.07 (0.89, 1.30)	0.469	1.37 (1.16, 1.64)	<0.001
Higher	1.38 (1.08, 1.77)	0.010	1.22 (0.96, 1.56)	0.105
Place of residence				
Urban	1.16 (0.99, 1.37)	0.073	1.08 (0.94, 1.26)	0.277
Rural				
Geographic region	Ref		Ref	
Barisal	1.01 (0.80, 1.27)	0.919	1.56 (1.26, 1.91)	<0.001
Chittagong	0.68 (0.55, 0.85)	0.001	1.20 (0.97, 1.47)	0.09
Dhaka	Ref		Ref	
Khulna	1.25 (0.99, 1.57)	0.058	1.26 (1.03, 1.52)	0.022
Rajshahi	1.02 (0.82, 1.26)	0.885	1.30 (1.06, 1.58)	0.011
Rangpur	1.41 (1.12, 1.78)	0.004	1.58 (1.28, 1.93	<0.001
Sylhet	0.67 (0.51, 0.89)	0.005	1.32 (1.05, 1.64)	0.016
Wealth index				
Poorest	Ref		Ref	
Poorer	1.18 (0.93, 1.49)	0.165	0.93 (0.77, 1.13)	0.471
Middle	1.18 (0.93, 1.48)	0.166	1.01 (0.83, 1.23)	0.915
Richer	1.37 (1.09, 1.72)	0.007	0.96 (0.78, 1.18)	0.687
Richest	1.84 (1.41, 2.40)	<0.001	1.11 (0.88, 1.39)	0.371
Body Mass Index				
Underweight	0.62 (0.52, 0.75)	<0.001	0.59 (0.50, 0.69)	<0.001
Normal weight	Ref		Ref	
Overweight	2.08 (1.71, 2.53)	<0.001	1.94 (1.68, 2.24)	<0.001
Obese	1.37 (1.13, 1.65)	0.001	2.26 (1.87, 2.73)	<0.001
Currently working				
No	Ref		Ref	
Yes	0.67 (0.54, 0.84)	0.001	0.90 (0.78, 1.05)	0.183
Diabetes				
No	Ref		Ref	
Yes	1.52 (1.26, 1.83)	<0.001	1.26 (1.08, 1.46)	0.003

Compared to male counterparts, females continued to have higher risk of hypertension in both surveys (AOR 1.42, 95% CI: 1.10, 1.82 in 2011 and AOR: 1.46, 95% CI: 1.26, 1.69 in 2018). Higher education and higher wealth status were significant factors for hypertension in 2011 but they had no significant effects on hypertension in 2018. Compared to the normal weight individuals the risk of hypertension among overweight and obese individuals were 2.08 and 1.37 times in 2011, and the risk changed to 1.94 times and 2.26 times in 2018, respectively. Individuals with diabetes were 52% (AOR: 1.52, 95% CI: 1.26, 1.83) and 26% (1.26, 95% CI: 1.08, 1.46) more likely to have hypertension in 2011 and 2018, respectively.

### Prevalence and risk factors of hypertension (18–34 years adults)

We analyzed younger adults (18–34 years) study participants separately for 2018 survey. Among younger adults, the overall prevalence of hypertension was 12.75% (95% CI: 11.77, 13.79) and the prevalence was higher in men (13.24%) than women (12.46%). In the logistic regression models for younger adults, there were no significant effects of sex, place of residence, marital status, education level, and working status in predicting hypertension. The key predictors of hypertension were age, geographic location, overweight/obesity, and diabetes. For example, adults in age group 25–29 had 50% (AOR: 1.50, 95% CI: 1.19, 1.89) higher likelihood of hypertension than the younger adults and odds of having hypertension among overweight and obese adults were 2.37 and 3.36 times compared to normal weight adults (Table 3).

**Table 3:** Unadjusted and adjusted odds ratios of potential factors associated with hypertension among adults age 34 and younger in Bangladesh, BDHS 2018.

Variables	Crude OR (95% CI)	p-value	Adjusted OR (95% CI)	p-value
Age group				
18–24	Ref		Ref	
25–29	1.61 (1.31, 1.98)	<0.001	1.50 (1.19, 1.89)	0.001
30–34	2.60 (2.10, 3.21)	<0.001	2.28 (1.79, 2.91)	<0.001
Sex				
Male	Ref		Ref	
Female	0.93 (0.78, 1.11)	0.438	0.82 (0.67, 1.02)	0.079
Marital status				
Not married	Ref		Ref	
Married	1.18 (0.96, 1.44)	0.114	0.82 (0.64, 1.06)	0.137
Educational level				
No education	Ref		Ref	
Primary	1.00 (0.67, 1.50)	0.987	1.00 (0.66, 1.53)	0.979
Secondary	1.02 (0.70, 1.48)	0.923	0.91 (0.61, 1.34)	0.642
Higher	1.19 (0.79, 1.78)	0.403	1.02 (0.66, 1.58)	0.87
Place of residence				
Urban	1.08 (0.90, 1.30)	0.406	1.00 (0.81, 1.24)	0.943
Rural	Ref		Ref	
Geographic region				
Barisal	1.37 (0.94, 1.99)	0.094	1.66 (1.1, 2.51)	0.019
Chittagong	1.68 (1.27, 2.21)	<0.001	1.84 (1.34, 2.52)	<0.001
Dhaka	Ref		Ref	
Khulna	1.32 (0.98, 1.80)	0.067	1.46 (1.02, 2.08)	0.045
Rajshahi	1.40 (0.98, 1.99)	0.043	1.68 (1.15, 2.45)	0.007
Rangpur	1.60 (1.21, 2.13)	0.001	2.16 (1.51, 3.08)	<0.001
Sylhet	1.18 (0.85, 1.64)	0.309	1.44 (0.99, 2.11)	0.068
Wealth index				
Poorest	Ref		Ref	
Poorer	1.52 (1.08, 2.15)	0.017	1.47 (1.02, 2.11)	0.038
Middle	1.31 (0.93, 1.84)	0.123	1.13 (0.78, 1.63)	0.515
Richer	1.82 (1.32, 2.51)	<0.001	1.67 (1.18, 2.38)	0.004
Richest	1.81 (1.31, 2.49)	<0.001	1.31 (0.98, 1.93)	0.130
Body Mass Index				
Underweight	0.74 (0.55, 1.00)	0.05	0.79 (0.59, 1.07)	0.133
Normal weight	Ref		Ref	
Overweight	2.48 (2.01, 3.05)	<0.001	2.35 (1.89, 2.93)	<0.001
Obese	3.67 (2.82, 4.76)	<0.001	3.24 (2.46, 4.28)	<0.001
Currently working				
No	Ref		Ref	
Yes	1.00 (0.84, 1.18)	0.984	0.86 (0.70, 1.07)	0.185
Diabetes				
No	Ref		Ref	
Yes	1.57 (1.20, 2.05)	0.001	1.50 (1.15, 1.94)	0.003

The contribution of predictor variables in explaining the socioeconomic status (SES) related inequality for having hypertension among adults 35 years and above during both the time-periods are presented in Table 4. In 2011, wealth index (46.58%), BMI (20.85%), place of residence (7.48%) contributed most to explain SES related inequalities for having hypertension among adults in Bangladesh. However, in 2018 the scenario changed a lot, the contribution of wealth index/ SES has reduced to 12.60% and the contribution of BMI has increased to 55.29%. The other most contributing factors to the inequality in 2018 are education level (10.00%), currently working (7.90%) and place of residence (6.34%).

**Table 4:** Estimates of decomposition analysis for contribution of various explanatory variables for hypertension among adults in Bangladesh, 2011 BDHS and 2018.

	2011						2018					
Variables	Coefficient	Elasticity	CI	Absolute contribution	Percent Contribution	%	Coefficient	Elasticity	CI	Absolute contribution	Percent Contribution	%
Age group						-0.802						0.105
35–44 (Ref)												
45–54	0.352***	0.119	0.017	0.002	1.45		0.34 ***	0.12	0.025	0.003	2.731	
55–64	0.523***	0.102	-0.005	0.000	-0.35		0.621***	0.176	-0.003	0	-0.443	
65–74	0.814***	0.102	-0.018	-0.002	-1.339		0.788***	0.127	-0.015	-0.002	-1.664	
75+	0.847***	0.067	-0.012	-0.001	-0.563		0.972***	0.086	-0.007	-0.001	-0.519	
Sex						1.027						-0.700
Male (Ref)												
Female	0.198***	0.115	0.012	0.001	1.027		0.233***	0.166	-0.005	-0.001	-0.7	
Marital status						-3.591						-4.197
Not married (Ref)												
Married	-0.205***	-0.199	0.025	-0.005	-3.591		-0.127**	-0.148	0.032	-0.005	-4.197	
Educational level						2.973						10.002
No education (Ref)												
Primary	-0.078*	-0.024	0.021	0.000	-0.359		0.066*	0.029	-0.073	-0.002	-1.89	
Secondary	0.034	0.007	0.216	0.001	1.072		0.192**	0.05	0.213	0.011	9.515	
Higher	0.191**	0.017	0.186	0.003	2.26		0.123*	0.015	0.182	0.003	2.377	
Place of residence (Ref)						7.481						6.340
Rural												
Urban	0.089**	0.024	0.433	0.010	7.481		0.051	0.018	0.397	0.007	6.34	
Geographic region						-3.051						-6.245
Barisal	0.008	0.001	-0.026	0.000	-0.01		0.27***	0.022	-0.048	-0.001	-0.958	
Chittagong	-0.224***	-0.043	0.044	-0.002	-1.361		0.11**	0.025	0.09	0.002	2.027	
Dhaka (Ref)												
Khulna	0.132**	0.02	0.023	0.000	0.34		0.137**	0.026	0.033	0.001	0.759	
Rajshahi	0.004	0.001	-0.038	0.000	-0.02		0.159**	0.031	-0.059	-0.002	-1.645	
Rangpur	0.204***	0.028	-0.099	-0.003	-2.005		0.28***	0.05	-0.143	-0.007	-6.32	
Sylhet	-0.227**	-0.015	0	0.000	0.005		0.165**	0.014	-0.009	0	-0.108	
Wealth index						46.581						12.596
Poorest (Ref)												
Poorer	0.093*	0.02	-0.32	-0.007	-4.742		-0.042	-0.012	-0.323	0.004	3.426	
Middle	0.094*	0.021	-0.024	-0.001	-0.374		0.009	0.003	0.012	0	0.028	
Richer	0.183**	0.044	0.311	0.014	9.864		-0.022	-0.006	0.299	-0.002	-1.504	
Richest	0.362***	0.087	0.661	0.058	41.833		0.064	0.018	0.653	0.012	10.646	
Body Mass Index						20.847						55.293
Underweight	-0.281***	-0.07	-0.194	0.014	9.853		-0.324***	-0.079	-0.196	0.016	13.885	
Normal weight (Ref)												
Overweight	0.434***	0.061	0.163	0.0100	7.187		0.405***	0.164	0.186	0.03	27.18	
Obese	0.175**	0.067	0.078	0.005	3.807		0.494***	0.091	0.174	0.016	14.228	
Currently working						4.113						7.903
No (Ref)												
Yes	-0.233***	-0.128	-0.044	0.006	4.113		-0.063	-0.057	-0.155	0.009	7.903	
Diabetes						2.251						5.472
No (Ref)												
Yes	0.246***	0.031	0.1	0.003	2.251		0.138***	0.037	0.166	0.006	5.472	

CI: concentration Index; Ref: Reference Category.

***p < 0.001;

**p < 0.05;

*p < 0.10.

### Sensitivity analysis

In our subgroup analysis by sex: age and BMI were significant predictors of hypertension across survey years. In the overall sample married individuals tend to have a lower risk of hypertension in both survey years, however; in the subgroup analysis we found that only married men in 2011 and married women in 2018 had a lower risk of hypertension compared to the unmarried counterparts, respectively. Working status was a significant predictor (AOR: 0.55, 95% CI: 0.39, 0.77) of hypertension among male only in 2011 [S1 Table].

The results of subgroup analyses by place of residence were consistent with the main results for age, wealth index, and body mass index in both survey years. However, the effect of gender, marital status, education, and diabetes status had differed between and within survey years. For example, female adults were found to have significantly higher hypertension risk in overall analysis in both survey years, however, there was no gender difference in hypertension risk in rural 2011 (AOR: 1.35,95% CI:0.98, 1.87) and urban 2018 (AOR:1.30, 95% CI: 0.99, 1.69) samples [S2 Table].

## Discussion

This is the first study to examine the changes in prevalence of hypertension and its risk factors among the adults in Bangladesh. A separate analysis was performed for two groups, 18–34 years as young adults and 35 years and above as older adults, using two waves (i. e. 2011 and 2018) of nationally representative survey data. The major findings are as follows: 1) in general, the prevalence of age-specific hypertension has increased and there is no subgroup in which it has decreased; 2) the prevalence has become almost double in some geographic locations, e.g. Chittagong, Sylhet; 3) among each wealth index category, there has been over 50% significant increase in the prevalence of hypertension; 4) regression models reveal that age and BMI were the independent risk factors of hypertension; 5) the adjusted effects of working status and wealth index are no longer significant in predicting the hypertension status in 2018; 6) trends are more favorable in women than in men, and in unmarried adults than in married adults 7) as expected, there are no differences in hypertension among younger adults (18–34 years) by sex, marital status, place of residence, and working status in 2018;and 8) the decomposition analysis shows that the major contributor of SES inequality of hypertension was wealth index (46.58%) in 2011, however in 2018 BMI (55.29%) contributed most to SES inequality of hypertension in Bangladesh.

Our study in line with previous studies identified that hypertension prevalence among adults in Bangladesh is increasing steadily [17, 19–22]. Comparing 2011 and 2018 data sets, it is evident that hypertension prevalence is increasing in almost all subgroups of adults in Bangladesh. From 2011 to 2018, the overall prevalence of hypertension among young adults aged 35 years or older significantly increased from 25.84% to 39.40%. The prevalence reached nearly double in 2018 among three of the seven geographic regions compared to 2011. The largest relative increase in prevalence of hypertension is in Chittagong and Sylhet followed by Barisal, Rajshahi, and Rangpur. Moreover, geographic location was found as a significant factor of hypertension even after controlling for other factors in the logistic regression models.

The prevalence of hypertension was high among females in both survey years; however, the relative increase among men was significantly higher (75%) than women (39%) from 2011 to 2018. Our findings of significant positive associations between adult hypertension status and established risk factors, such as age and BMI, are similar to other studies [15, 17, 33–35]. The odds of having hypertension among overweight and obese adults are 94% and 126% higher than normal-weight adults, respectively in 2018 and these results are also consistent with studies in Bangladesh and in other developing countries [15, 22, 34, 36, 37]. The prevalence of hypertension increased by more than 50% among non-diabetic individuals in Bangladesh. Diabetic status was significantly associated with hypertension in both the 2011 and 2018 surveys.

Higher education and higher socio-economic status were significantly associated with hypertension only in 2011. The adjusted odds ratio for the richest individuals dropped from 1.84 in 2011 to 1.11 in 2018. The adjusted odds of having hypertension were not significantly different among adults having no education, primary education, and secondary education in 2011. However, the reverse situation is prevalent in 2018. Individuals having secondary education have higher odds of having hypertension than individuals with no education. The likelihoods of having hypertension among the poorest, poorer and middle-income groups were not found significant in both periods. However, richer, and richest adults had 37% and 84% more odds of having hypertension than the poorest adults in 2011.

In the decomposition analysis we found that, wealth index was the important factor contributing to the hypertension inequality in 2011 whereas in 2018, BMI has been observed as the most contributing factor to the inequality of hypertension among adults aged 35 years and above in Bangladesh. Bangladesh’s economic situation is improving, and the disparity between the middle and upper classes may be narrowing; additionally, in recent years, people have had greater access to unhealthy lifestyles, including unhealthy food consumption, as a result of rising obesity [38–40]. This could explain why the wealth index’s influence on hypertension has decreased while BMI’s influence is increasing.

Another unique aspect of this study in Bangladesh is the identification of contributing factors associated with hypertension among younger adults (18–34 years). Hypertension prevalence in this age group was 12.75% with a slightly higher prevalence in urban adults than rural adults. Like older adults (≥35 years), age, overweight and obesity and being diabetic are significant risk factors for hypertension among young adults. Younger adults’ greater likelihood of having hypertension being living in other than Dhaka regions, particularly in Rangpur and Chittagong, are plausibly associated with their lower access to the socioeconomic development opportunities, poverty and geographical remoteness [11, 41]. The risk of hypertension among adults aged 25–29 and 30–34 years are 1.5 and 2.28 times, respectively than adults aged 18–24 years. Interestingly, the odds of having hypertension is 1.5 times higher [AOR: 1.50, 95% CI: 1.15, 1.94] among diabetic younger adults. Hypertension prevalence among active age population is a concern considering its economic and health burden in Bangladesh [39, 40, 42–45].

In the subgroup analysis by sex, we found that education level was not significantly associated with the hypertension among women in 2011 and 2018. This is expected as men and women have equal opportunities for access to education over the last two-three decades. Overtime, the likelihood of having hypertension among obese females have increased more than five times compared to normal-weight female after adjusting other covariates. To better understand the relationship between obesity and hypertension among Bangladeshi women further studies are required. Marital status among men was found insignificant in 2011 though, the odds of having hypertension is 52% lower among married men in 2018 and, the situation is reversed among female overtime.

In the subgroup analysis by place of residence (i.e. urban vs rural), the odds of hypertension between male and female were significantly different in rural areas in 2011 whereas this difference is only significant among urban areas in 2018. Urban females had 1.64 times higher odds of having hypertension than urban males in 2011 whereas sex is not a significant factor for hypertension among urban adults in 2018. In contrast, there was no significant difference in having hypertension among rural male and female in 2011 while female in rural areas have 1.54 times higher likelihood of hypertension than male in rural areas in 2018. This may be related to the socio-economic development in rural areas where people are getting facilities like urban areas.

### Strengths and limitations

One of the greatest strengths of this study is the use of large sample which represents adults 18 years and older population in Bangladesh. To our knowledge, this study for the first time estimated the national hypertension prevalence and its risk factors among general adults of Bangladesh. The second important strength is that we compared changes in estimates of hypertension predictors between 2011 and 2018 surveys along with subgroups analyses: such as 18–34 years, by sex, and by place of residence. Thirdly, we considered complex survey data analysis methodology in our study which produces variance estimation accounting for post-stratification adjustments to the sampling weights. Finally, we used Wagstaff decomposition technique to find the factors contributing towards the change between two survey periods. However, some of the important factors such as physical activity, dietary behavior, drinking habits are not included as those variables are not collected by BDHS. Moreover, considering the nature of the cross-sectional survey design, we are unable to comment on the causal relationships between the outcome and the predictors.

Despite these limitations, this large-scale population-based study may help us to conclude that almost 4 in 10 adults (35+ years) have high blood pressure and the relative increment in prevalence over time is alarming among men (74%) than women (39%). Age and overweight/obesity are the two most important risk factors of hypertension for all adult population irrespective of sex, residence, educational attainment, and wealth index. The risk of hypertension varies from one geographic location to other, with higher risks in Rangpur and Barisal division and relatively lower risk in Chittagong division as compared to Dhaka division suggested the importance of further study to examine higher increase overtime in these regions. Rapid increase in obesity, less physical activity, unplanned urbanization may be the driving forces for the extensive burden of hypertension in Bangladesh. In light of our study findings and increasing rates of mobile phone users, social media campaign and mobile phone text message may be directed for informing young and middle age adults about the importance of healthy lifestyle and physical activity to avoid the saddle of hypertension and other non-communicable diseases.

## Supporting information

S1 Table Associations between potential risk factors and hypertension status of adults age 35 and older by gender and survey year in Bangladesh, BDHS 2011–2018. (DOCX)

S2 Table Associations between potential risk factors and hypertension status of adults age 35 and older by place of residence and survey year in Bangladesh, BDHS 2011–2018. (DOCX)
