# Supplementary material for: Changes in prevalence and risk factors of hypertension among adults in Bangladesh: An analysis of two waves of nationally representative surveys
Source: PLoS One. 2021 Dec 2;16(12):e0259507. doi: 10.1371/journal.pone.0259507 (PMC8638884; doi:10.1371/journal.pone.0259507)
Supplement: S1 Table — (DOCX) [file pone.0259507.s001.docx]

| **TABLE S1.** Associations between potential risk factors and hypertension status of adults age 35 and older by gender and survey year in Bangladesh, BDHS 2011-2018. | | | | | | | | |
| --- | --- | --- | --- | --- | --- | --- | --- | --- |
|  | Male 2011 BDHS | | Male 2018 BDHS | | Female 2011 BDHS | | Female 2018 BDHS | |
| Variables | OR (95% CI) | p-value | OR (95% CI) | p-value | OR (95% CI) | p-value | OR (95% CI) | p-value |
| Age group |  |  |  |  |  |  |  |  |
| 35-44 | Ref |  | Ref |  | Ref |  | Ref |  |
| 45-54 | 1.79 (1.39, 2.31) | <0.001 | 1.8 (1.45, 2.24) | <0.001 | 1.81 (1.44, 2.29) | <0.001 | 1.70 (1.38, 2.09) | <0.001 |
| 55-64 | 2.13 (1.61, 2.80) | <0.001 | 3.04 (2.41, 3.85) | <0.001 | 2.47 (1.8, 3.38) | <0.001 | 2.51 (2.01, 3.13) | <0.001 |
| 65-74 | 3.44 (2.48, 4.77) | <0.001 | 4.52 (3.49, 5.85) | <0.001 | 4.23 (2.99, 5.98) | <0.001 | 2.85 (2.07, 3.93) | <0.001 |
| 75+ | 3.25 (2.11, 5.02) | <0.001 | 5.45 (3.65, 8.14) | <0.001 | 4.69 (3.06, 7.17) | <0.001 | 4.28 (2.82, 6.49) | <0.001 |
| Marital status |  |  |  |  |  |  |  |  |
| Not married | Ref |  | Ref |  | Ref |  | Ref |  |
| Married | 0.77 (0.46, 1.28) | 0.313 | 0.48 (0.32, 0.74) | 0.001 | 0.77 (0.64, 0.94) | 0.01 | 0.88 (0.72, 1.07) | 0.211 |
| Educational level |  |  |  |  |  |  |  |  |
| No education | Ref |  | Ref |  | Ref |  | Ref |  |
| Primary | 0.98 (0.75, 1.26) | 0.853 | 1.31 (1.05, 1.63) | 0.017 | 0.83 (0.68, 1.02) | 0.084 | 0.99 (0.82, 1.19) | 0.925 |
| Secondary | 1.08 (0.81, 1.44) | 0.585 | 1.66 (1.29, 2.13) | <0.001 | 1.13 (0.85, 1.5) | 0.406 | 1.11 (0.88, 1.40) | 0.377 |
| Higher | 1.75 (1.24, 2.46) | 0.001 | 1.65 (1.22, 2.22) | 0.001 | 0.89 (0.58, 1.39) | 0.614 | 0.68 (0.45, 1.01) | 0.054 |
| Place of residence |  |  |  |  |  |  |  |  |
| Urban | 1.08 (0.84, 1.38) | 0.546 | 1.06 (0.84, 1.32) | 0.639 | 1.21 (0.99, 1.49) | 0.068 | 1.12 (0.94, 1.35) | 0.203 |
| Rural | Ref |  | Ref |  | Ref |  | Ref |  |
| Geographic region |  |  |  |  |  |  |  |  |
| Barisal | 1.01 (0.71, 1.44) | 0.951 | 1.44 (1.06, 1.96) | 0.02 | 0.99 (0.75, 1.32) | 0.947 | 1.70 (1.3, 2.22) | <0.001 |
| Chittagong | 0.76 (0.54, 1.08) | 0.126 | 1.06 (0.77, 1.46) | 0.723 | 0.62 (0.48, 0.82) | 0.001 | 1.31 (1.03, 1.68) | 0.027 |
| Dhaka | Ref |  | Ref |  | Ref |  | Ref |  |
| Khulna | 1.37 (0.98, 1.92) | 0.069 | 1.18 (0.88, 1.58) | 0.259 | 1.17 (0.89, 1.55) | 0.256 | 1.32 (1.05, 1.67) | 0.017 |
| Rajshahi | 1.04 (0.74, 1.47) | 0.825 | 1.31 (0.97, 1.76) | 0.076 | 0.96 (0.74, 1.23) | 0.733 | 1.3 (0.99, 1.71) | 0.058 |
| Rangpur | 1.67 (1.19, 2.36) | 0.004 | 1.49 (1.1, 2.04) | 0.011 | 1.25 (0.92, 1.7) | 0.161 | 1.66 (1.26, 2.17) | <0.001 |
| Sylhet | 0.86 (0.56, 1.31) | 0.476 | 1.21 (0.89, 1.66) | 0.229 | 0.57 (0.4, 0.82) | 0.003 | 1.39 (1.06, 1.83) | 0.018 |
| Wealth index |  |  |  |  |  |  |  |  |
| Poorest | Ref |  | Ref |  | Ref |  | Ref |  |
| Poorer | 1.16 (0.81, 1.65) | 0.427 | 0.98 (0.74, 1.29) | 0.861 | 1.19 (0.9, 1.57) | 0.212 | 0.89 (0.7, 1.14) | 0.353 |
| Middle | 1.25 (0.87, 1.78) | 0.224 | 0.99 (0.74, 1.33) | 0.963 | 1.12 (0.84, 1.5) | 0.431 | 1.02 (0.8, 1.31) | 0.866 |
| Richer | 1.33 (0.93, 1.9) | 0.116 | 0.96 (0.72, 1.29) | 0.793 | 1.34 (1.02, 1.78) | 0.039 | 0.95 (0.72, 1.24) | 0.703 |
| Richest | 1.70 (1.13, 2.58) | 0.012 | 1.35 (0.97, 1.89) | 0.078 | 1.91 (1.39, 2.61) | <0.001 | 0.94 (0.71, 1.26) | 0.701 |
| Body Mass Index |  |  |  |  |  |  |  |  |
| Underweight | 0.56 (0.44, 0.71) | <0.001 | 0.58 (0.46, 0.73) | <0.001 | 0.7 (0.53, 0.92) | 0.011 | 0.6 (0.47, 0.76) | <0.001 |
| Normal weight | Ref |  | Ref |  | Ref |  | Ref |  |
| Overweight | 1.84 (1.41, 2.39) | <0.001 | 2.1 (1.69, 2.61) | <0.001 | 2.6 (1.87, 3.62) | <0.001 | 1.79 (1.49, 2.17) | <0.001 |
| Obese | 2.36 (1.65, 3.39) | <0.001 | 1.84 (1.34, 2.54) | <0.001 | 1.31 (1.02, 1.69) | 0.037 | 2.56 (2.01, 3.27) | <0.001 |
| Currently working |  |  |  |  |  |  |  |  |
| No | Ref |  | Ref |  | Ref |  | Ref |  |
| Yes | 0.55 (0.39, 0.77) | <0.001 | 0.93 (0.7, 1.25) | 0.642 | 0.77 (0.58, 1.02) | 0.066 | 0.88 (0.74, 1.04) | 0.127 |
| Diabetes |  |  |  |  |  |  |  |  |
| No | Ref |  | Ref |  | Ref |  | Ref |  |
| Yes | 1.20 (0.89, 1.6) | 0.226 | 1.14 (0.92, 1.42) | 0.218 | 1.78 (1.4, 2.26) | <0.001 | 1.35 (1.1, 1.66) | 0.004 |
