# Supplementary material for: Changes in prevalence and risk factors of hypertension among adults in Bangladesh: An analysis of two waves of nationally representative surveys
Source: PLoS One. 2021 Dec 2;16(12):e0259507. doi: 10.1371/journal.pone.0259507 (PMC8638884; doi:10.1371/journal.pone.0259507)
Supplement: S2 Table — (DOCX) [file pone.0259507.s002.docx]

| **TABLE S2.** Associations between potential risk factors and hypertension status of adults age 35 and older by place of residence and survey year in Bangladesh, BDHS 2011-2018. | | | | | | | | |
| --- | --- | --- | --- | --- | --- | --- | --- | --- |
|  | Urban 2011 BDHS | | Urban 2018 BDHS | | Rural 2011 BDHS | | Rural 2018 BDHS | |
| Variables | OR (95% CI) | p-value | OR (95% CI) | p-value | OR (95% CI) | p-value | OR (95% CI) | p-value |
| Age group |  |  |  |  |  |  |  |  |
| 35-44 | Ref |  | Ref |  | Ref |  | Ref |  |
| 45-54 | 1.74 (1.32, 2.3) | <0.001 | 1.76 (1.37, 2.26) | <0.001 | 1.89 (1.53, 2.35) | <0.001 | 1.77 (1.45, 2.16) | <0.001 |
| 55-64 | 2.75 (1.88, 4.02) | <0.001 | 2.9 (2.2, 3.81) | <0.001 | 2.39 (1.85, 3.09) | <0.001 | 2.77 (2.3, 3.33) | <0.001 |
| 65-74 | 3.99 (2.48, 6.41) | <0.001 | 3.83 (2.53, 5.8) | <0.001 | 4.09 (3.17, 5.27) | <0.001 | 3.64 (2.86, 4.63) | <0.001 |
| 75+ | 3.44 (2.01, 5.9) | <0.001 | 5.25 (3.08, 8.98) | <0.001 | 4.45 (3.24, 6.12) | <0.001 | 5.06 (3.66, 6.99) | <0.001 |
| Sex |  |  |  |  |  |  |  |  |
| Male | Ref |  | Ref |  | Ref |  | Ref |  |
| Female | 1.64 (1.14, 2.35) | 0.008 | 1.3 (0.99, 1.69) | 0.055 | 1.35 (0.98, 1.87) | 0.066 | 1.54 (1.29, 1.83) | <0.001 |
| Marital status |  |  |  |  |  |  |  |  |
| Not married | Ref |  | Ref |  | Ref |  | Ref |  |
| Married | 0.87 (0.62, 1.2) | 0.389 | 0.75 (0.56, 1.01) | 0.056 | 0.68 (0.56, 0.82) | <0.001 | 0.83 (0.68, 1.02) | 0.077 |
| Educational level |  |  |  |  |  |  |  |  |
| No education | Ref |  | Ref |  | Ref |  | Ref |  |
| Primary | 0.97 (0.7, 1.36) | 0.872 | 1.5 (1.17, 1.94) | 0.002 | 0.85 (0.71, 1.02) | 0.083 | 1.03 (0.88, 1.21) | 0.721 |
| Secondary | 1.17 (0.88, 1.57) | 0.276 | 1.35 (1.01, 1.81) | 0.042 | 1.02 (0.8, 1.32) | 0.846 | 1.43 (1.15, 1.77) | 0.001 |
| Higher | 1.37 (0.97, 1.93) | 0.075 | 1.19 (0.8, 1.77) | 0.383 | 1.46 (0.99, 2.15) | 0.053 | 1.34 (0.98, 1.83) | 0.068 |
| Geographic region |  |  |  |  |  |  |  |  |
| Barisal | 0.77 (0.51, 1.18) | 0.233 | 1.57 (0.97, 2.54) | 0.065 | 1.09 (0.83, 1.43) | 0.548 | 1.57 (1.25, 1.98) | <0.001 |
| Chittagong | 0.85 (0.62, 1.15) | 0.290 | 1.35 (0.98, 1.86) | 0.066 | 0.66 (0.49, 0.88) | 0.004 | 1.15 (0.88, 1.51) | 0.299 |
| Dhaka | Ref |  | Ref |  | Ref |  | Ref |  |
| Khulna | 1.24 (0.93, 1.65) | 0.143 | 1.07 (0.77, 1.48) | 0.688 | 1.28 (0.96, 1.73) | 0.095 | 1.32 (1.04, 1.66) | 0.02 |
| Rajshahi | 1.06 (0.75, 1.49) | 0.745 | 1 (0.71, 1.41) | 0.998 | 1.03 (0.79, 1.34) | 0.816 | 1.40 (1.1, 1.77) | 0.006 |
| Rangpur | 1.63 (1.2, 2.22) | 0.002 | 1.4 (1, 1.98) | 0.053 | 1.41 (1.06, 1.87) | 0.017 | 1.65 (1.31, 2.1) | <0.001 |
| Sylhet | 0.88 (0.57, 1.34) | 0.542 | 1.05 (0.76, 1.46) | 0.763 | 0.65 (0.46, 0.92) | 0.014 | 1.4 (1.07, 1.82) | 0.013 |
| Wealth index |  |  |  |  |  |  |  |  |
| Poorest | Ref |  | Ref |  | Ref |  | Ref |  |
| Poorer | 0.91 (0.39, 2.09) | 0.818 | 0.78 (0.48, 1.27) | 0.315 | 1.2 (0.94, 1.53) | 0.151 | 0.96 (0.78, 1.18) | 0.679 |
| Middle | 1.14 (0.6, 2.13) | 0.692 | 1.32 (0.87, 2.01) | 0.191 | 1.19 (0.93, 1.53) | 0.159 | 0.96 (0.77, 1.2) | 0.721 |
| Richer | 1.74 (1.03, 2.92) | 0.037 | 1.13 (0.75, 1.69) | 0.567 | 1.33 (1.03, 1.73) | 0.03 | 0.92 (0.72, 1.17) | 0.478 |
| Richest | 2.43 (1.44, 4.09) | 0.001 | 1.18 (0.79, 1.75) | 0.419 | 1.62 (1.15, 2.28) | 0.006 | 1.13 (0.86, 1.5) | 0.383 |
| Body Mass Index |  |  |  |  |  |  |  |  |
| Underweight | 0.56 (0.38, 0.83) | 0.004 | 0.49 (0.34, 0.71) | <0.001 | 0.64 (0.52, 0.79) | <0.001 | 0.61 (0.51, 0.72) | <0.001 |
| Normal weight | Ref |  | Ref |  | Ref |  | Ref |  |
| Overweight | 1.81 (1.28, 2.56) | 0.001 | 1.82 (1.41, 2.35) | <0.001 | 2.28 (1.79, 2.9) | <0.001 | 2.00 (1.69, 2.38) | <0.001 |
| Obese | 1.33 (0.94, 1.89) | 0.111 | 2.53 (1.86, 3.44) | <0.001 | 1.37 (1.09, 1.72) | 0.007 | 2.15 (1.68, 2.73) | <0.001 |
| Currently working |  |  |  |  |  |  |  |  |
| No | Ref |  | Ref |  | Ref |  | Ref |  |
| Yes | 0.69 (0.48, 0.99) | 0.044 | 0.87 (0.67, 1.13) | 0.299 | 0.67 (0.5, 0.88) | 0.004 | 0.90 (0.75, 1.08) | 0.243 |
| Diabetes |  |  |  |  |  |  |  |  |
| No | Ref |  | Ref |  | Ref |  | Ref |  |
| Yes | 1.54 (1.16, 2.04) | 0.003 | 1.18 (0.93, 1.49) | 0.175 | 1.51 (1.19, 1.93) | 0.001 | 1.31 (1.08, 1.59) | 0.005 |
